# Parallel DNA Extraction From Whole Blood for Rapid Sample Generation in Genetic Epidemiological Studies

**DOI:** 10.3389/fgene.2020.00374

**Published:** 2020-04-29

**Authors:** Kiara Lee, Anubhav Tripathi

**Affiliations:** Center for Biomedical Engineering, School of Engineering, Brown University, Providence, RI, United States

**Keywords:** microfluidics, NGS, genomic DNA, magnetic beads, solid-phase extraction, whole blood, genetic epidemiological study, point of care (POC)

## Abstract

Large-scale genetic epidemiological studies require high-quality analysis of samples such as blood or saliva from multiple patients, which is challenging at the point of care. To expand these studies’ impact, minimal sample storage time and less complex extraction of a substantial quantity and good purity of DNA or RNA for downstream applications are necessary. Here, a simple microfluidics-based system that performs genomic DNA (gDNA) extraction from whole blood was developed. In this system, a mixture of blood lysate, paramagnetic beads, and binding buffer are first placed into the input well. Then, the gDNA-bound paramagnetic beads are pulled using a magnet through a central channel containing a wash buffer to the output well, which contains elution buffer. The gDNA is eluted at 55°C off the chip. The 40-minute microfluidic protocol extracts gDNA from six samples simultaneously and requires an input of 4 μL of diluted blood and a total reagent volume of 75 μL per reaction. Techniques including quantitative PCR (qPCR) and spectrofluorimetry were used to test the purity and quantity of gDNA eluted from the chip following extraction. Bead transport and molecular diffusional analysis showed that an input of less than 4 ng of gDNA (∼667 white blood cells) is optimal for on-chip extraction. There was no observable transport of inhibitors into the eluate that would greatly affect qPCR, and a sample was successfully prepared for next-generation sequencing (NGS). The microfluidics-based extraction of DNA from whole blood described here is paramount for future work in DNA-based point-of-care diagnostics and NGS library workflows.

## Introduction

Genetic testing from various starting samples such as blood, urine, and saliva is becoming more popular due to the rise in next-generation sequencing (NGS). Genetic epidemiological studies allow for greater possibilities in personalized medicine, but to be more accessible, sample collection should be adapted to the point of care (Seyerle and Avery, 2013). Point-of-care (POC) devices are devices that allow biological tests to be performed on-site, not requiring expensive equipment and/or mobility of the samples. For a genetic epidemiological study, this would signify the preparation the of the sample for NGS on-site, reducing the need for shipment and storage of biological samples. However, isolation and preparation of DNA samples for sequencing can be time-consuming.

DNA extraction has evolved from using harsh chemicals such as chloroform to a method called solid phase extraction (SPE) (Ali et al., 2017). Solid-phase extraction is based on liquid and solid phases, with DNA (or RNA) being adsorbed onto the solid phase depending on the pH and salt concentrations of the buffers used. After the DNA is adsorbed onto the solid phase, it must be washed and eluted off. Various solids for adsorption have been used in SPE and typically involve centrifugation, vacuum filtration, or column separation during the wash and elution steps (Tan and Yiap, 2009). These processes during the wash and elution steps increase experimental time and require additional equipment. Advances in SPE methods have allowed for the use of magnetic beads as the solid phase. DNA binds to the magnetic beads, and they are washed with wash buffers by pipetting, which removes the need for centrifugation, vacuum filtration, or column separation. To elute the DNA off the beads, the DNA-bound beads are simply placed into an elution solution, leaving the isolated DNA in the supernatant.

Whole blood is a common biological starting sample for DNA extraction. Compared to other minimally invasive sources of genomic gDNA (gDNA), such as saliva or buccal cells, gDNA yield is higher and less fragmented (Koshy et al., 2017). Whole blood contains red blood cells (RBCs), white blood cells (WBCs), platelets, and plasma, with gDNA found in the nuclei of WBCs. gDNA found in blood is of high quality and is used in forensics, cancer diagnoses, and various other biological tests. Previously, there a few POC have been devices created that use magnetic beads to extract gDNA from whole blood (Duarte et al., 2010; Gong and Li, 2014; Hung et al., 2015). These POC devices each use small, handheld tools that handle microliter-scale volumes, known as microfluidic devices. They also utilize magnetic beads for purification and highlight gDNA extraction efficiencies. The yield is sufficient for preparation for an Illumina^®^ Sequencing platform, which requires as little as 1ng. In each of these studies, however, there is a lack of an description of the extracted gDNA solution’s purity, which would indicate its suitability for downstream use in NGS. More quantitative methods, such as quantitative PCR (qPCR) can be used to observe inhibition, or contamination, in amplified samples. To obtain accurate NGS data, the DNA samples must be as free from contamination as possible (Gruber, 2015). Contamination can arise from a variety of sources such as bacteria and inhibitors of polymerase chain reaction (PCR), which is an integral part of DNA sequencing. In blood, hemoglobin, heme, and anticoagulants such as EDTA and Heparin may inhibit PCR efficiency, as may the reagents used in DNA extraction protocols (Schrader et al., 2012).

This paper proposes a simple, automated microfluidics-based system that performs gDNA extraction from human whole blood. A solution of blood lysate, binding buffer, and DNA-bound paramagnetic beads are added to a microfluidic chip, where the beads are moved using a magnet through a wash buffer and end, in an elution buffer. The microfluidic chip reduces the number of wash steps needed compared to a manual protocol. This system has several advantages compared to those previously developed. Firstly, the polyvinyl alcohol magnetic particles (M-PVA Magnetic Beads) being used have low, unspecific protein binding properties, high functionalization potential, and high magnetite content. These factors contribute to maximizing the purity of the DNA eluate and the speed of extraction. Additionally, these magnetic beads do not require the use of chaotropic salts for nucleic acid binding (Oster et al., 2001). Chaotropic salts, which are typically present in lysing and binding buffers during SPE are PCR inhibitors, so the reduction of their presence increases the likelihood of successful PCR (Sur et al., 2010). The system is simple to use, utilizes small sample and reagent volumes, does not require continuous flow, and can perform multiple extractions in parallel. The microfluidic protocol is based on diffusion principles and can be automated but can easily be performed manually, signifying that the DNA extractions can be done in the absence of electrical equipment. The lack a need for electricity increases the accessibility of the device to a variety of settings, such as at the point of care. Overall, the microfluidic chip described here extracts gDNA of high quality and purity from whole blood, which has the potential to significantly reduce the time and cost of sample generation for genetic epidemiological studies.

## Materials and Methods

### Human Whole Blood

Human male whole blood was purchased from Golden West Biosolutions (Temecula, CA, United States). The blood samples were stored in 5-mL tubes, with either Sodium Heparin or Sodium EDTA anticoagulants. The samples were tested for common infectious diseases by Gold West Biosolutions and were received one day after the bleed date. All samples were stored at 4°C.

### Off-Chip Workflow

**FIGURE 1:**
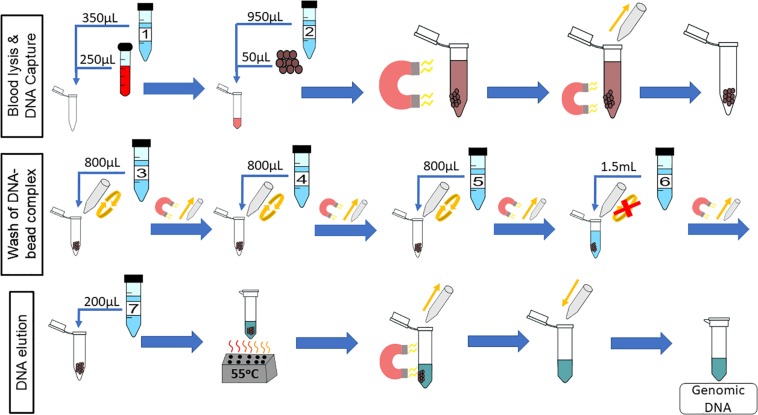
Off-chip Workflow, a graphic of the chemagic^TM^ DNA Blood250 Kit protocol. A 250-μL sample of blood is lysed, and then magnetic beads and a binding buffer are added to the solution to bind the gDNA. The gDNA-bound magnetic beads are moved to the side of the tube using a magnet, and the supernatant is removed. The gDNA-magnetic bead complex is washed four times and an elution buffer is added. The gDNA elutes off of the beads at 55°C, and the supernatant contains the gDNA of interest.

The chemagic^TM^ DNA Blood250 Kit (PerkinElmer, Waltham, MA, United States) is a manual gDNA extraction kit that was converted for on-chip use. Unless otherwise stated, all reagents used are from that kit. The workflow for this kit is depicted in Figure 1, which will be referred to as “off-chip.” First, 250 μL of whole blood is mixed with 350 μL of Lysis Buffer 1 and incubated for 5 min at room temperature. Then, 50 μL of magnetic beads and 950 μL of Binding Buffer 2 are added to the lysed blood cells, and these are mixed and incubated for 5 min at room temperature. The tube containing lysed blood cells and the DNA-bound magnetic beads is placed onto a magnetic rack to separate the DNA-bound magnetic beads from the lysate for 2 min at room temperature. The supernatant is discarded, and 800 μL of Wash Buffer 3 is added to the tube; the beads are resuspended, and the tube is placed back onto the magnetic rack for 1 min. The supernatant is discarded, and the washing procedure is repeated using Wash Buffers 4 and 5. After Wash Buffer 5 is removed, the tube is left in the magnetic rack, and 1.5 mL of Wash Buffer 6 is added without disrupting the pellet. This is left for 90 s, and the supernatant is discarded. Then, 200 μL of Elution Buffer 7 is mixed with the DNA-bound magnetic beads and incubated for 10 min at 55°C for DNA elution. The tube is then placed on to the magnetic rack, and the beads are separated from the eluate. The eluate contains purified DNA. The expected yield is 5–10 μg of genomic DNA from normal healthy whole blood.

### Determination of DNA Yield

Spectrofluorometry was performed based on guidelines from ThermoFisher Scientific Inc. (Waltham, MA, United States) using the Quant-iT^TM^ PicoGreen^TM^ dsDNA Assay Kit. Signal measurements of all samples were performed using the EnVision 2105 Multimode Plate Reader (PerkinElmer, Waltham, MA, United States). Subsequent linear regression analysis was performed in Microsoft Excel (Microsoft, Redmond, WA, United States). For all experiments, the standard curve used for linear regression had *R*^2^ > 0.99.

### Microfluidic Chip Fabrication

The PDMS-glass microfluidic chip has two rows of six microfluidic separators per chip that are spaced 9 mm apart, such that DNA extraction can be performed from six individual blood samples simultaneously with a multichannel pipette.

The wells of the device were formed by curing the polydimethylsiloxane (PDMS) in a sandwich mold composed of an SU-8 master mold to form the microfluidic features and a machined aluminum mold to form the wells of the devices (Supplementary Figure S1). The two molds are aligned and held together with spring clamps. Uncured PDMS is then poured into the top opening into the gap between both molds. Once cured at 70°C, the film of PDMS formed in the gap is released from both molds. The wells have been formed by the aluminum mold, but a thin layer of PDMS occludes them, which is hole-punched out. The PDMS film is then bonded to a 75 × 50 mm glass slide. This method allows for more precise well formation when compared to conventional PDMS hole-punching methods.

### Magnet Movement

To remove variability in the magnet’s movement when performing experiments, an in-house device was used to standardize magnet motion. The device consists of an x-stage for left-right motion. The stage is a linear screw-drive stage (igus^®^ plastics for longer life^®^, Providence, RI, United States) with a stepper motor used to control the stage motion (Applied Motion Products, Inc., Watsonville, CA, United States) based on an in-house script. The magnet is a 2″ × 1/2″ × 1/4″ thick neodymium bar magnet (Grade N42, Item #BY084), with a surface field of 3424 Gauss purchased from K & J Magnetics, Inc. (Pipersville, PA, United States).

### Magnetic Force Modeling

Modeling was performed with COMSOL Multiphysics (Burlington, MA, United States).

### Primers for Real-Time PCR

Real-time PCR was performed on the CFX96 Touch Real-Time PCR Detection System (Bio-Rad Laboratories, Inc., Hercules, CA, United States). TaqMan^TM^ Gene Expression Assay ID# Hs00243216_s1, a primer for human sex-determining region Y (SRY) gene was used (ThermoFisher Scientific Inc., Waltham, MA, United States).

The Taqman^TM^ Gene Expression Master Mix (ThermoFisher Scientific Inc., Waltham, MA, United States) was used for the assay.

### Pure Genomic DNA

Human genomic DNA (male) was obtained from Promega Corporation (Madison, WI, United States).

### Next Generation Sequencing Preparation

Extracted DNA samples were prepared for Illumina^®^ Sequencing using the NEXTFLEX^®^ Rapid DNA-Seq Kit (PerkinElmer, Waltham, MA, United States). For analysis, the Agilent DNA 1000 Kit was used on the Agilent 2100 Bioanalyzer device (Agilent Technologies, Santa Clara, CA, United States).

### Statistical Analysis

Statistical analyses were performed in GraphPad Prism 8 (San Diego, CA, United States). ^∗^*p* ≤ 0.05, ^∗∗^*p* ≤ 0.01, ^∗∗∗^*p* ≤ 0.001, and ^****^p ≤ 0.0001.

## Results and Discussion

### Reduced Blood Volume for Translation to the Chip

One of the goals of the microfluidic chip was to reduce the number of wash steps needed in the gDNA extraction protocol. To identify one wash buffer, or combination of wash buffers, the off-chip protocol was performed only using one wash step with one wash buffer per experiment. Wash Buffer 3 was found to have comparable DNA yield and purity to the original protocol (data not shown).

The volumes of the remaining chemagic^TM^ protocol reagents needed to be scaled down significantly, as the depth of the wells in the microfluidic chip is ∼70 μL. Various linear scale-downs of the off-chip protocol were tested meaning each reagent was scaled down by the same factor. The best results were found via scaling the starting volume of blood from 250 to 4 μL, and therefore all reagents were scaled linearly by a factor of 62.5. Thus, Lysis Buffer 1 was scaled to 5.6 μL, Binding Buffer 2 to 15.2 μL, and the magnetic beads to 0.8 μL. Together, this volume of 25 μL constitutes the input to the microfluidic chip. The output is the eluate containing Elution Buffer 7, and the scaling factor made the required volume 3.2 μL. However, this volume would be too small to be pipetted from the microfluidic chip for elution, and since the microfluidic chip is based on diffusion, this stark difference in volume between the input and output would cause the input to diffuse into the output well. To (1) maintain similar volumes between the input and output and (2) not overdilute the gDNA eluted such that the concentration would be difficult to quantify, an elution volume of 16 μL was used, which makes the solution five times more dilute than to that of the full protocol.

**FIGURE 2:**
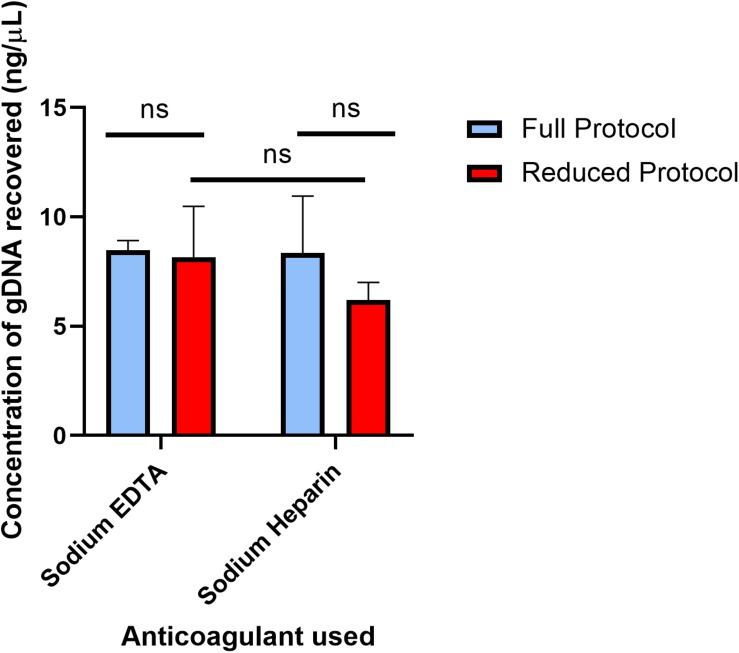
Concentration of gDNA from starting whole blood volumes of 250 and 4 μL off-chip. The concentrations of gDNA extracted under the full protocol and reduced protocol were compared in two different anti-coagulated whole blood samples. The full protocol starting blood volume is 250 μL, while the reduced protocol starting blood volume is 4 μL. To compare the two anticoagulants, multiple unpaired *t*-tests were performed. The mean difference in recovery between the two protocols (Sodium EDTA difference = 0.31, SE = 1.16 and Sodium Heparin difference = 2.14, SE = 1.37) was not significantly greater than 0 [Sodium EDTA: *t*(5) = 0.27, two-tail adjusted *p* = 0.80; Sodium Heparin: *t*(6) = 1.57, two-tail adjusted *p* = 0.31], providing evidence that either anticoagulant type can be used in the gDNA extraction protocol. To compare the mean gDNA recovery of the two protocols, as well as the anticoagulant types, two-way ANOVA was performed (α = 0.05). The mean difference between Sodium EDTA and Sodium Heparin was not significantly greater than 0 (*p* = 0.28). The mean difference between the full protocol and the reduced protocol was not significantly greater than 0 (*p* = 0.21). Lastly, using Sidak’s multiple comparisons test, there was no statistically significant difference between Sodium EDTA and Sodium Heparin using the reduced protocol (*p* = 0.32). Together, these results provide evidence that neither the anticoagulant type nor the starting blood volume has a significant effect on the gDNA yield.

The full protocol starting with 250 μL of blood and the 4 μL reduced blood volume protocol were each performed off-chip, and the results are compared in Figure 2 to indicate whether off-chip gDNA yield was similar between the two protocols. The full protocol was performed 2 days after the bleed date of the donor, and the reduced protocol was performed 4 days after the full protocol. As mentioned previously, since the elution volume for the reduced volume protocol is 5 times more dilute than that of the full, off-chip protocol, the concentration of DNA eluted using the reduced protocol was multiplied by 5 for comparison purposes. Following the original protocol, EDTA-anticoagulated blood yielded 8.46 ng/μL, and Heparin-anticoagulated blood yielded 8.35 ng/μL. Using the reduced protocol, EDTA-anticoagulated blood yielded 8.15 ng/μL, and Heparin-anticoagulated blood yielded 6.20 ng/μL. There was no statistically significant difference between the yields of the original protocol and the reduced protocol for either anticoagulant type. Thus, it was concluded that the reduced protocol, using a starting blood volume of 4 μL, was appropriate for translation onto the microfluidic chip.

The expected yield for the chemagic^TM^ protocol is 25–50 ng/μL, but the yield here with both protocols when quantified with spectrofluorometry was approximately 8 ng/μL. Using spectrometry via Nanodrop^TM^ (ThermoFisher Scientific Inc., Waltham, MA, United States), the extracted gDNA samples were measured to be at the expected concentration, but due to the presence of residual reagents, the Nanodrop^TM^ concentration values are easily variable. Thus, only spectrofluorometry was used as the quantification technique for gDNA concentration here. Other studies have compared the use of these two quantification techniques and also reported lower concentration measurements using spectrofluorometry (Nakayama et al., 2016).

Additionally, DNA yield from blood can vary dramatically, even from samples with a similar white blood cell count. The wide error bars are likely a result of this, which contributes to the lack of a statistically significant difference between the Heparin samples’ protocols. In addition, the delay between gDNA extraction of the samples plays a role in the lower recovery of the reduced protocol samples (Bulla et al., 2016). However, EDTA acts as a DNA stabilizer, and therefore it is expected that DNA concentration will remain similar despite delays in extraction and blood collection. The anticoagulant type was not significant for this study but was explored in order to investigate the applicability to human blood of Kotikalapudi and Patel’s (2015) work, which compared the two anticoagulants’ effects on long-term cattle DNA extraction and storage (Kotikalapudi and Patel, 2015). Similarly, they concluded that the anticoagulant type was not significant for DNA extraction and storage.

### The Microfluidic Chip

**FIGURE 3:**
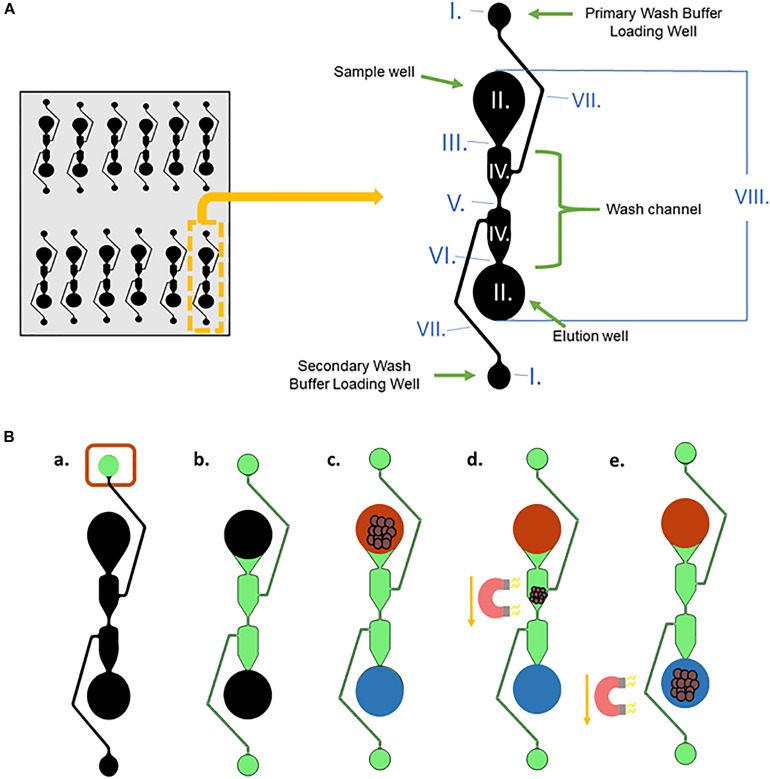
DNA extraction from whole blood procedure. **(A)** A single microfluidic separator with labeled well and channel names, along with the dimensions of the chip. The entire length of one separator is 15.5 mm, and they are spaced 9mm apart horizontally. The channels have a depth of 150 μm, and the sample well, elution well, and wash buffer loading wells are 7.65 mm in depth. I. Wash Buffer Loading Wells, radii = 0.75 mm; II. Sample well and elution well: radii = 1.75 mm; III. Width = 0.212 mm; IV. Width = 1.517 mm; V. Width = 0.134 mm; VI. Width = 0.148 mm; VII. Width = 0.125 mm; VIII. Length = 12 mm. **(B)** (a,b) Wash Buffer 3 is loaded into the Primary Wash Buffer Loading Well and diffuses through the center Wash Channel to the Secondary Wash Buffer Loading Well. (c) The input solution of lysed blood and DNA-bound magnetic beads and output solution, Elution Buffer 7, are added simultaneously to the sample well and elution well, respectively. (d,e) A magnet moves the DNA-bound magnetic beads from the sample well through the wash buffer into the elution well.

One of the microfluidic separators and its dimensions, well, and channel names are shown in Figure 3A. The purpose of a separator is to simplify the number of wash steps needed for magnetic bead-based DNA extraction from blood. To reduce magnetic bead aggregation in the corners of each segment as the beads move through the chip, the well is angled inward. The wash channel has an hourglass shape to reduce hydrodynamic flow. This also slows the magnetic beads, allowing them to be more effectively washed.

Figure 3B depicts the procedure for liquid loading and magnet movement for the microfluidic chip-based gDNA extraction. In Figure 3B, black represents empty wells and channels before loading, and they are filled as follows. First, 20 μL of Wash Buffer 3 (green) is loaded into the Primary Wash Buffer Loading Well (Figure 3Ba) and moves via capillary action through the center Wash Channel to the Secondary Wash Buffer Loading Well (Figure 3Bb). The wash buffer does not seep into the Sample Well or the Elution Well due to the capillary flow-controlled Wash Channel. Then, the input solution (red), which is the 25 μL volume obtained after lysing the blood and binding the gDNA to the magnetic beads (using the Reduced Protocol from Figure 2), and 16 μL of Elution Buffer 7 (blue) are simultaneously loaded into the Sample Well and the Elution Well, respectively (Figure 3Bc). The two solutions stay separated due to the length of the wash channel, which contributes to the slow diffusion time between the two wells. A magnet is used to move the magnetic beads from the Sample Well, through the Wash Channel, and into the elution well (Figures 3Bd,e).

#### Magnet Movement for Pulling Beads Through the Chip

**FIGURE 4:**
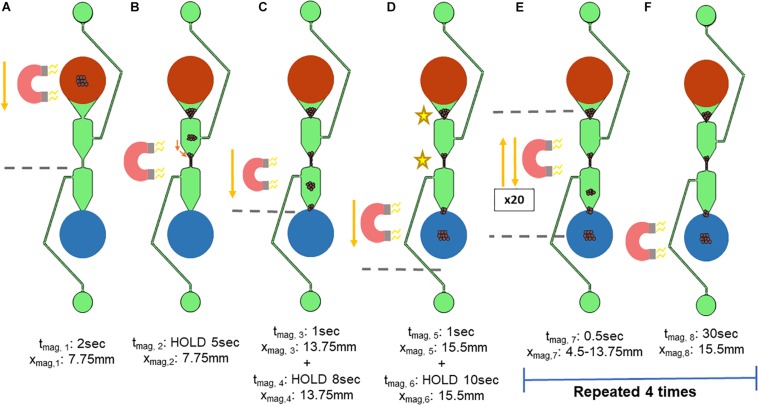
Standardized magnet movement. The movement of the x-stage holding the microfluidic chip over a magnet was based on an in-house script. The variable x_mag_ refers to the location on the microfluidic separator in which the edge of the bar magnet is located, with the top of the sample well (shown in red) being x_mag_ = 0 mm. t_mag_ refers to the length of time that the x-stage is moving. **(A,B)** The x-stage then moves the microfluidic chip 7.75 mm (x_mag,__1_) over 2 s (t_mag,__1_) such that the magnet is beneath the center of the microfluidic chip. **(B)** The x-stage pauses (x_mag,__2_) for 5 s (t_mag,__2_) to allow the magnetic beads to move toward the magnet. **(C)** The x-stage moves for 1 s (t_mag,__3_) to 13.75 mm the length of the separator (x_mag,__3_), pausing at the start of the elution well for 8 s (x_mag, 4_ and t_mag,__4_). **(D)** The x-stage moves for 1 s (t_mag,__5_) past the elution well to 15.5mm the length of the microfluidic separator (x_mag,__5_) and pauses to concentrate the magnetic beads into the elution well for 10 s (x_mag, 6_ and t_mag,__6_). The x-stage must move the microfluidic chip repeatedly such that the magnet is beneath two key aggregation points (indicated by the stars), at the narrowest parts of the microfluidic separator. **(E,F)** To encourage movement of the magnetic beads at these locations toward the elution well, the x-stage cycles between the dotted lines, which are x_mag,__7_ = 4.5 mm and x_mag,__8_ = 13.75 mm. One cycle consists of: (1) movement away from the elution well, and (2) movement toward the elution well, followed by a 0.5 s pause. Each cycle was repeated 20 times, followed by a 30-s pause (t_mag,__8_). These two steps were repeated 4 times.

Once the appropriate volumes for the microfluidic chip were established, the reduced protocol was transferred to the microfluidic chip. The movement of the magnet necessary for on-chip extraction was first investigated manually, via moving a magnet beneath the microfluidic chip by hand. To standardize, this movement was automated using the x-stage of an in-house device. The microfluidic chip is placed onto the x-stage and moved over a magnet, which is depicted in Figure 4. The movement was first optimized for use with magnetic beads that were not bound to gDNA, and then modified for those that were.

The movement of the x-stage begins with the magnet beneath the sample well to first concentrate the magnetic beads to the bottom of the sample well (Figure 4A). The variable x_mag_ refers to the location on the microfluidic separator in which the edge of the bar magnet is located, with the top of the sample well (shown in red) being x_mag_ = 0 mm and the bottom of the elution well (blue) being x_mag_ = 15.5 mm. t_mag_ refers to the length of time that the x-stage is moving or holding at a position (HOLD). The x-stage then moves the microfluidic chip such that the magnet is beneath the center of the microfluidic chip (Figures 4A,B). Here, the x-stage holds for 5 s to allow the magnetic beads to move toward the magnet (Figure 4B). Next, the x-stage moves for 1 s down the length of the separator, holding at the start of the elution well for 8 s (Figure 4C). Then, the x-stage moves past the elution well for 1 s and holds to concentrate the magnetic beads into the elution well for 10 s (Figure 4D). The magnetic beads were found to aggregate in two major points in the wash channel, indicated by the stars in Figure 4D. Thus, the magnetic field needed to be concentrated at these two points, but not intensify aggregation. The magnet was therefore moved repeatedly between the two aggregation points for 20 cycles, as shown in Figure 4E. One cycle consists of (1) brief (less than 0.5 s) movement away from the elution well, and (2) brief movement toward the elution well followed by a 0.5-s pause. This movement allows the magnetic beads that are trapped in the upper part of the microfluidic chip to be attracted to the magnet while ensuring that the magnetic beads are spending more time being pulled toward the elution well. Lastly, to gather the magnetic beads fully into the elution well, the magnet is held at the bottom edge (x_mag,__8_ = 15.5 mm) of the elution well for 30 s to ensure complete movement of the magnetic beads to the elution well (Figure 4F). The reduction of aggregation cycles and the 30-s hold at the elution well were then repeated 4 times, a number that was determined by observation to maximize the number of beads moving through the chip.

Once the magnet motions are complete the contents of the elution well are removed from the microfluidic chip and placed into a separate tube and incubated for 10 min at 55°C. The tube is then placed onto a magnetic rack, and the beads separated from the eluate. The eluate contains purified gDNA.

### gDNA Reduction for Successful Movement Through the Microfluidic Chip

Initial results showed scarce amounts of gDNA recovered from the microfluidic chip due to there being little to no movement of the magnetic beads from the aggregation points to the elution well. Various experiments were done to isolate the cause of this, including magnetic force calculations on the glass slide using varying magnet strengths varying the volume of magnetic beads, and performing extraction with a pure gDNA sample to remove the influence of blood’s many components. It was found that the magnetic beads’ movement was significantly altered by the presence of pure gDNA. Without gDNA present, the magnetic beads moved quickly through the chip, but when gDNA was bound to the beads, they aggregated at the aggregation points (Figure 4D) and there was remarkably less movement through these points. It was concluded that the starting amount of gDNA in the starting sample well heavily influenced the ability of the magnetic beads to move to the elution well. The use of a stronger magnet was considered, and modeling showed that the magnetic force would increase by 10N (Supplementary Figure S2) if the supplier’s strongest magnet of similar dimensions was used (the current magnet being used is the second strongest). However, this slight increase of magnetic force would cause the magnetic beads to more quickly and forcefully aggregate, increasing the difficulty of overcoming the DNA-DNA interactions. The amount of starting gDNA needed to be reduced.

Anticoagulated whole blood samples were diluted in either nuclease-free water or 1X PBS to a final volume of 4 μL, and gDNA was extracted using the reduced protocol. It was found that there was no significant difference between diluting blood in 1XPBS and in nuclease-free water in terms of the percent recovery of gDNA on- and off-chip (data not shown). Nuclease-free water may not be available to all users of this device, so 1XPBS was used for dilution of the blood.

#### Dilution of Blood to Various Starting Amounts to Characterize the Starting Dilution Needed to Maximize gDNA Recovery

**FIGURE 5:**
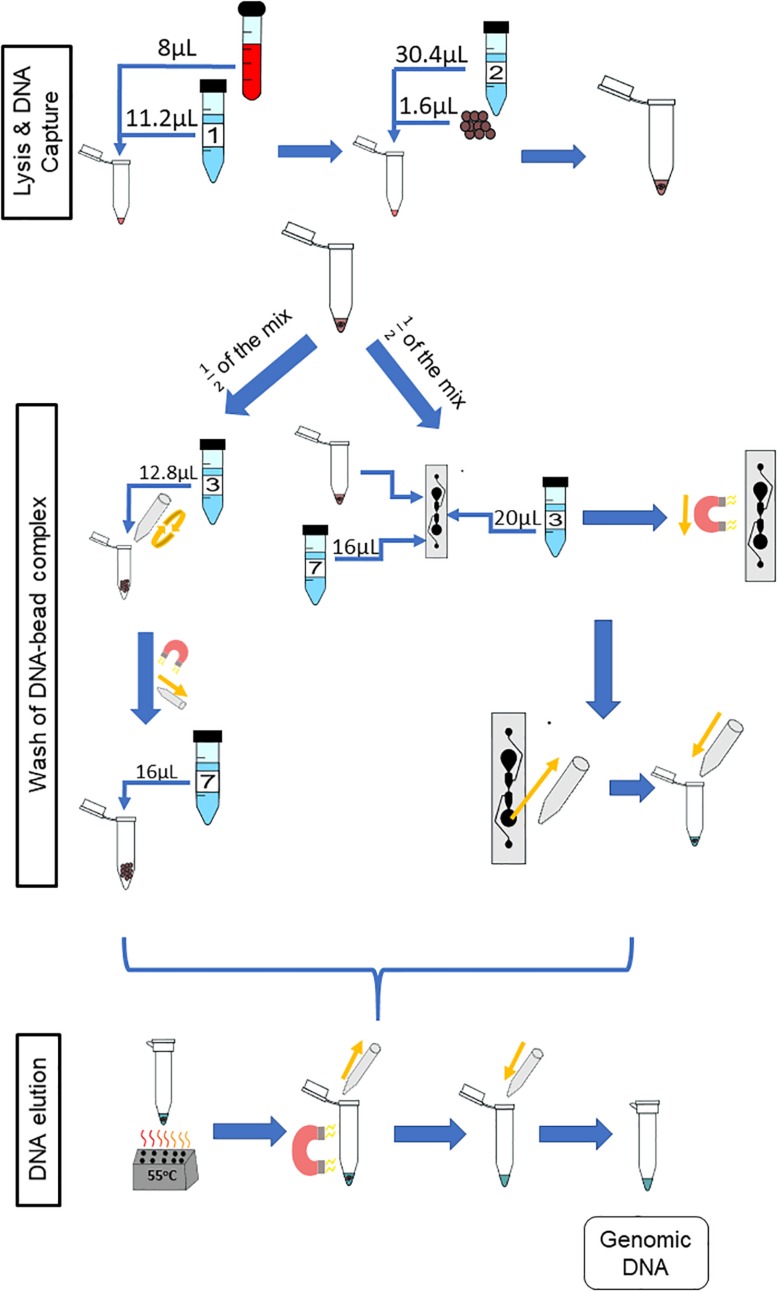
Paired protocol to compare gDNA extraction off- and on-chip. The starting volume of the sample was doubled to 8 from 4 μL so as to have a starting sample that would be split equally between the two protocols for comparison purposes. The reagents were scaled accordingly. Half of the starting sample (diluted blood, lysing buffer, binding buffer and magnetic beads) was used with the off-chip protocol **(left)** and the other half with the on-chip protocol **(right)**.

**FIGURE 6:**
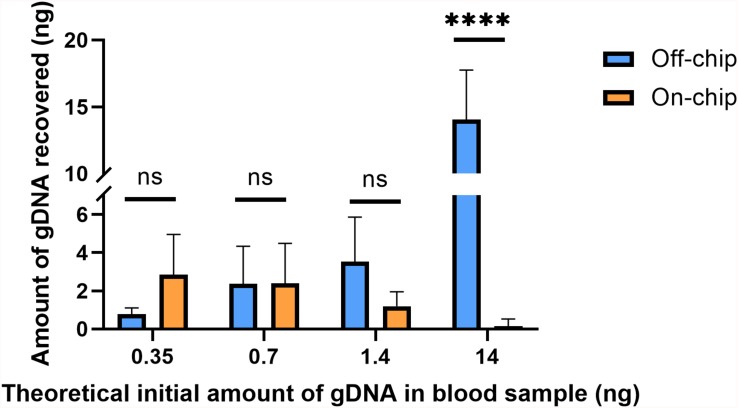
Amount of gDNA extracted on and off-chip with varying dilutions of blood. Whole blood was diluted to various starting amounts to compare the recovery of gDNA on-chip to that off-chip. The undiluted starting amount (14 ng) of gDNA in whole blood was found using the off-chip 4 μL protocol. The subsequent dilutions were simply divided by their dilution factors to yield the values in the *x*-axis. Two-way ANOVA (α = 0.05) was performed to compare off-chip versus on-chip and *n* = 6. One sample from the positive control, 14 ng off-chip, was excluded as an outlier due to significantly low yield. Dunnett’s multiple comparisons test was used for deeper analysis, and 14 ng/μL, *p* < 0.0001 (SE of difference = 1.183) and all others *p* > 0.2.

To better understand the restraints of the microfluidic chip with respect to the amount of gDNA in the starting sample, various dilutions of whole blood were tested. The dilutions were done off-chip and on-chip and compared. To reduce sample variability between the off- and on-chip protocols, both started from the same starting sample. Stock solutions of whole blood wer diluted 1:10 and 1:20 in 60 μL of 1X PBS, and 1:40 in 80 μL of 1X PBS. From there, the initial starting sample was made in one tube at two times the on-chip protocol volume, with 8 μL of the diluted blood. All other reagents were scaled accordingly. Six separate replicates of each stock dilution were placed into separate tubes. For each, the solution made after the binding step was divided equally into two for on-chip protocol and off-chip protocol washing (Figure 5); the starting amount of gDNA for each protocol was from a 4-μL sample and eluted in 16 μL of Elution Buffer 7. The yields from both protocols at the various dilutions were compared. Figure 6 shows the amount of gDNA recovered from various dilutions of whole blood. It should be noted that the blood samples leading to the results in Figures 2 and 6 are not from the same donor. Additionally, the amount recovered shown in the graph does not take into account the dilution factor of the elution volume, unlike Figure 2. For these experiments, Sodium Heparin anticoagulated blood was used, and the extractions occurred 9 days after the donor’s bleed date.

Using the Picogreen^TM^ assay, the average gDNA yield in the undiluted blood sample was measured to be ∼14 ng off-chip, and the average amount of DNA recovered on-chip was 0.15 ng. The dilutions theoretically should theoretically be starting amounts of 1.4, 0.7, or 0.35 ng and on-chip they recovered an average of 1.2, 2.38, and 2.85 ng, respectively. As the blood sample becomes more diluted, i.e., a lower starting amount of gDNA, the on-chip protocol and off-chip protocol recover similar amounts of gDNA, with the on-chip protocol surpassing that of the off-chip at the highest dilution factor. The samples have similarly sized error bars, which is most likely due to the uneven distribution of white blood cells in the samples, which will cause increased variability in the gDNA present in the starting sample between replicates.

The theoretical initial amounts on the x-axis of Figure 6 were calculated by dividing the undiluted off-chip sample’s yield by the solutions’ respective dilution factors; these may not be their true starting amounts. To better compare the off-chip and on-chip yields at the various dilutions, a percent recovery [% recovery(i)] was calculated by comparing the on-chip protocol to the off-chip protocol at that specific sample’s amount (Table 1). This was done as an alternative to comparing all values to the undiluted starting amount, which originated from a different stock solution to the others. The percent recovery indicates that there is a point, below 0.7 ng at which the on-chip protocol captures more gDNA than the off-chip protocol. A similar trend was seen with another data set (data not shown). The trend in gDNA recovery on-chip is such that lower amounts of starting gDNA led to improved recovery of the gDNA. To further investigate this, along with the significant increase in percent recovery for both protocols at lower amounts of starting gDNA, mathematical modeling was performed.

**TABLE 1:** Percent recovery off-chip and on-chip of various diluted blood samples.

Sample Starting Amount (ng)	% Recovery
14	1.06%
1.4	40%
0.7	101%
0.35	366%

$$\%recovery\left(i\right)=\frac{onchipextractedamount\left(i\right)}{offchipextractedamount\left(i\right)}\times 100\%$$

where *i* = specified sample’s starting amount.

#### Mathematical and Mechanistic Understanding of Magnetic Bead Transport

For this gDNA extraction technique to be successful in general, the magnetic beads must (1) bind the gDNA and (2) be transported in the presence of a magnetic field. The reagent protocol for binding the gDNA off-chip and on-chip was unchanged, and was thus not considered for modeling purposes. For bead transport, the original protocol uses tubes, so bead movement is largely unrestricted. Here, and in other microfluidic devices, the magnetic beads must be transported through more restrictive and complex geometries.

**FIGURE 7:**
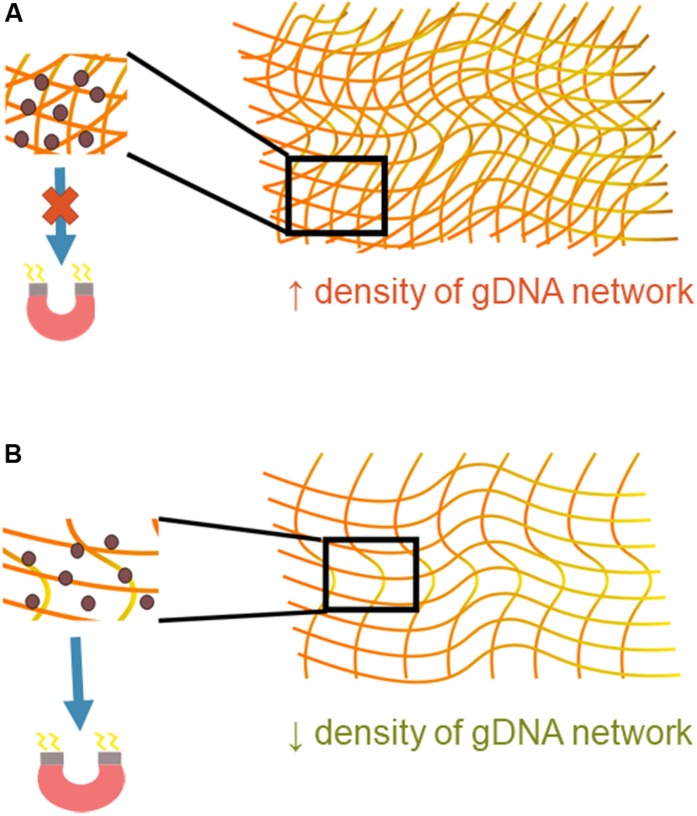
Mechanistic understanding of bead transport. **(A)** A higher density of gDNA means there are more opportunities for interaction between DNA molecules, increasing the presence of intermolecular forces. These forces are likely greater than that of the magnetic force, which causes inhibition of the gDNA-magnetic bead complex’s movement through the microfluidic chip. **(B)** A lower density of gDNA signifies fewer opportunities for interaction between gDNA molecules. The magnetic force is likely greater than the intermolecular forces between the gDNA-gDNA networks, allowing the gDNA-magnetic bead complex to move more freely through the microfluidic chip.

The amount of gDNA present in the starting sample was shown to have a significant effect on bead transport. gDNA is very long, can interact with itself, and exists in a variety of topologies (Mirkin, 2001). Once bound, magnetic beads are introduced into the multiple gDNA strand complex, and then together they must be transported under the influence of a magnet. The gDNA-magnetic bead complex can be thought of as a deformable cluster of complicated geometrical shapes. The deformability of the cluster depends on the geometry through which it is moving but, more importantly, also depends on the magnetic force felt by the paramagnetic beads and the number and strength of the interaction of DNA molecules. Understanding of the physics behind interactions between DNA molecules is complex and largely theoretical; however, it is known that such interactions are present when the molecules are in close proximity in an aqueous solvent (Li et al., 2014). More gDNA present in this system that means there is a higher density of the gDNA networks and more opportunities for intermolecular forces between gDNA molecules (Figure 7). The higher density causes the magnetic beads to be entangled in the gDNA network, with the intermolecular forces between molecules overpowering the magnetic force, negatively affecting the beads’ transport through the intricate geometry. With less gDNA present the opposite appears to be true; the magnetic force is greater than that of the intermolecular forces between gDNA molecules, causing movement of the gDNA-magnetic bead complex through the microfluidic chip.

To look at the relationship between gDNA density and the magnetic beads more simply, the relationship between the available surface area of the spherical beads and the surface area of gDNA was investigated. Using a hemocytometer, the concentration of the magnetic beads (C_bead_) was measured to be approximately 23 × 10^6^ beads/μL, and their diameters are 1.5 μm. The total available surface area for a given volume [V_bead_(x)] of magnetic beads can therefore be calculated.

Totalavailablemagneticbeadsurfacearea

$$\;=SA_{bead}\times C_{bead}\times V_{bead}\left(x\right)$$

where *x* is dependent upon the initial sample volume. Here, the bead volume was kept constant for all dilutions; therefore, the total available magnetic bead surface area was found to be 1.31 cm^2^.

Theoretical values were used to find the total available surface area for gDNA in the system. The concentration of leukocytes in fresh, whole blood was used to find the number of cells in a given volume, N_cells_, of which, on average, there are 5,500 leukocytes/μL (Blumenreich, 1990). There are 6.4 billion base pairs in the human genome (Piovesan et al., 2019), and the size of extracted gDNA fragments once purified is 100–200 kbp (DNA Extraction and Purification, 2013). Here, it will be assumed that the gDNA present in the system is already in the ∼150 kbp fragments. Using these parameters, the number of fragments present in one white blood cell can be calculated, F_gDNA_.

For modeling purposes, the gDNA fragments were assumed to be spherical, using the calculated radius of gyration of ∼0.9 μm (Department of Chemistry, 2005; Tree et al., 2013) to calculate the surface area of one gDNA fragment (SA_gDNA_).

$$TotalavailablegDNASA=N_{cells}\left(y,z\right)\times F_{gDNA}\times SA_{gDNA}$$

where $F_{gDNA}=\frac{humangenomelength}{sizeofextractionfragments}$ and the number of cells, *N*_cells_ is dependent upon *y*, the volume of blood used and *z*, the dilution factor of the blood.

From these two equations, the ratio of DNA surface area to bead surface area can be calculated:

$$gDNA:beadratio=\beta =\frac{TotalavailablegDNASA}{TotalavailablemagneticbeadSA}$$

Variables *x*, *y*, and *z* were varied to estimate the gDNA:bead ratio (β) at the various dilution factors for which results are shown in Figure 6 (Table 2).

**TABLE 2:** gDNA:bead ratio (β) calculations for varying dilutions of whole blood.

Dilution factor	Undiluted blood volume [μL]	N_cells_	Total gDNA surface area available [cm^2^]	β
None	4	22,000	100	76
1:10	0.4	2,200	10	7.6
1:20	0.2	1,100	5	3.8
1:40	0.1	550	2.5	1.9

β is significant for successful movement of the gDNA-magnetic bead complex through the microfluidic chip. For the undiluted sample, β = 76, and once diluted, β decreases by a factor of 10. This indicates that there is a critical ratio of β < 10 at which the transport of magnetic beads in a gDNA complex improves, because this correlates with the findings shown in Figure 6 that gDNA extraction is improved with blood dilution. The number of beads in a given area increases with the dilution factor. A larger number of beads in an area allows a greater force to be applied to the DNA networks to separate them for transport through the microfluidic chip. This estimation of β is not precise, as gDNA topology varies heavily in cells. However, the trend is what is significant for this study, which indicates the importance of the gDNA:bead ratio in the successful movement of the gDNA-bead complex through the microfluidic chip.

### Quantitative PCR of gDNA Extracted From Diluted Blood Samples

**FIGURE 8:**
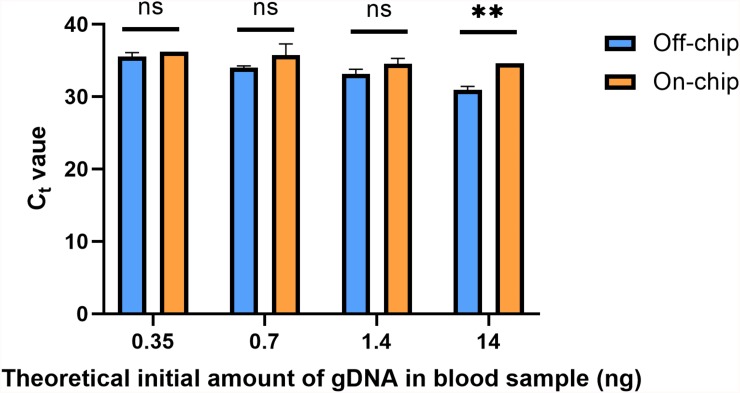
C_t_ values of gDNA extracted from diluted blood samples. qPCR using Taqman chemistry was performed on the gDNA samples from Figure 6. Samples that did not have a C_t_ value in the 40-cycle PCR were omitted; these were two on-chip samples from 14 ng, two on-chip samples from 0.35 ng, and one on-chip sample from 0.7 ng. Data were analyzed using two-way ANOVA (α = 0.05) and Sidak’s multiple comparisons test for further analysis, *n* = 3. For 14 ng, *p* = 0.004 and all others, *p* > 0.091. Standard curve using 5-fold dilutions: *R*^2^ = 0.9993, slope = –3.32.

A downstream preparation step for NGS is PCR. To investigate the purity and quality of the diluted gDNA samples, quantitative PCR (qPCR) was performed using Taqman probes for the human SRY gene. The average C_t_ value for three selected samples from each of the starting amounts shown in Figure 6 is shown in Figure 8. Replicates 2, 3, and 5 from each experimental group were selected for qPCR analysis at random. In Figure 8, the eluate of on-chip and off-chip extractions from the undiluted sample and the diluted samples were not significantly different when compared. However, replicates that did not amplify within the 40-cycle PCR were excluded from the graph; these were two on-chip samples from 14 ng, two on-chip samples from 0.35 ng, and one on-chip sample from 0.7 ng. For the replicates that did not amplify, their extracted gDNA amounts were cross-checked with data from Figure 6. One of the excluded 14 ng replicates did not have gDNA recovered on-chip, matching its C_t_ value. For all the other excluded values, however, the replicates did have sufficient gDNA recovered yet did not amplify. This indicated the presence of inhibition due to a component in the eluate on-chip. This was further investigated.

#### PCR Inhibition From Wash Buffer 3

PCR inhibition can arise from a variety of sources in this system. Anticoagulated blood has PCR inhibitors such as heme and Heparin (Al-Soud and Rådström, 2001). The protocol does not involve a Proteinase K digestion; therefore it is unlikely that heme is contributing to the inhibition (Akane et al., 1994). Heparin inhibits ribonucleases, which is also not relevant here (Wang et al., 1992). Carryover of blood lysate components like proteins is possible, but is unlikely due to the negative charge of the M-PVA beads (Oster et al., 2001). Though the contents of the reagents used are proprietary, general knowledge about their contents provided the possibility of them containing PCR inhibitors.

The volume of Elution Buffer 7 put into the elution well is 16 μL and the sample volume input onto the chip is 25 μL. Since this microfluidic chip is based on diffusion, the difference in volume between the two wells could cause hydrodynamic flow, causing Wash Buffer 3 to flow into the elution well. Additionally, when the eluate is removed from the microfluidic chip, it is pipetted up and down to gather all the magnetic beads. There is a sudden change in volume, which could cause the sample well contents to quickly flow into the wash channel, causing the wash buffer to flow into the elution well. Wash buffers typically contain ethanol, which has been shown to inhibit PCR (Huggett et al., 2008).

**FIGURE 9:**
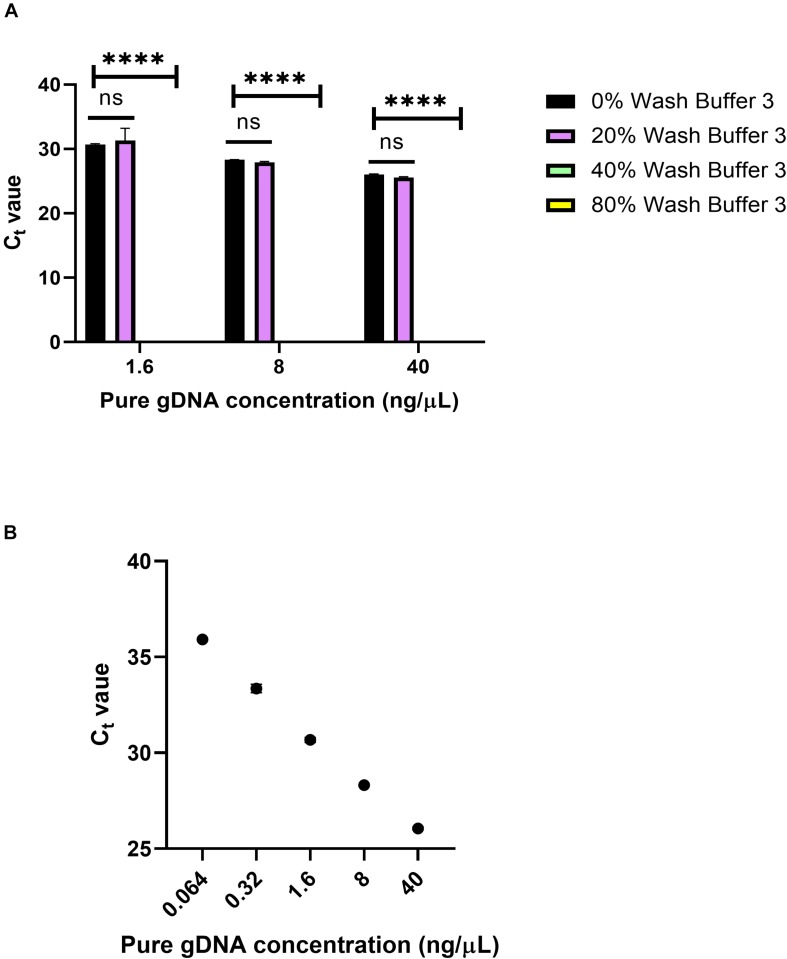
Wash Buffer 3 inhibition. **(A)** C_t_ values from qPCR of 0, 20, 40, and 80% solutions of Wash Buffer 3 with pure genomic DNA solutions and *n* = 1 with three replicates. Statistical significance was found by two-way ANOVA (α = 0.05), using Dunnett’s multiple comparisons test for deeper analysis. Within each concentration, C_t_ values were compared to 0% Wash Buffer or a pure solution of gDNA. *P*-values were <0.0001 for all comparisons. **(B)** Standard curve for panel **(A)** using 5-fold dilutions. *R*^2^ = 0.9973, slope = –3.543.

To see the effects of Wash Buffer 3 on qPCR, varying percentages of Wash Buffer 3 were added to pure gDNA solutions. Pure human male gDNA was used to eliminate the possibility of inhibition from blood components. First, 10 μL solutions of 1.6, 8, and 40 ng/μL of pure gDNA were made, containing either 0, 20, 40 or 80% Wash Buffer 3 (Figure 9). Then, 1 μL of this solution was used in the qPCR reaction, using the same Taqman probes as in Figure 8. For all solutions, 20% of Wash Buffer 3 did not significantly inhibit the reaction. However, above 20% of Wash Buffer 3 none of the solutions amplified within the 40-cycle PCR. It was concluded that small amounts of Wash Buffer 3 ingress into the eluate greatly inhibits qPCR.

##### Reduction of Wash Buffer Ingress into the Elution Well

Wash Buffer 3 fills the wash channel via capillary action and does not enter into the sample or elution wells as it fills. Therefore, the source of the contamination had to be due to wash buffer ingress into the elution well via a different phenomenon. Analysis with colored liquids showed that Wash Buffer 3 ingresses into the elution well when the eluate is removed for elution, as hypothesized. To reduce this effect, two changes were made to the on-chip protocol. Firstly, the elution buffer volume was increased to 32 μL. This was done to reverse the direction of diffusion such that some of the elution buffer will diffuse into the wash channel instead of vice versa. Secondly, to remove the eluate from the elution well for the off-chip heated elution, volumes in the sample well and elution well were removed simultaneously (just as they were added in simultaneously) such that equal volumes remained after removal. Therefore, when the eluate is pipetted up and down, there is no volume difference to cause the sample well contents to flow into the elution well. With these changes, the sample well has 23 μL of solution and the elution well has 32 μL of solution. A volume of 20 μL is removed from the sample well and a volume of 28 μL is removed from the elution well, respectively. The sample volume removed is discarded, and the eluate is placed into a separate tube for the heated elution.

**FIGURE 10:**
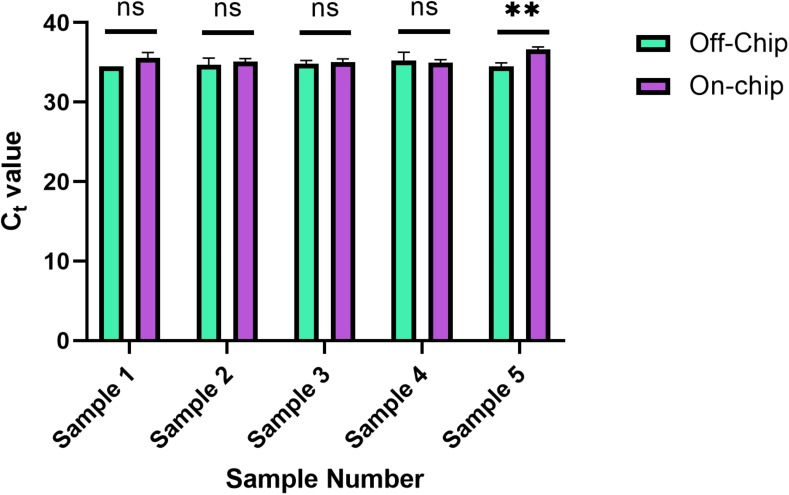
Reduced ingress of Wash Buffer 3 C_t_ values. C_t_ values obtained for five separate diluted blood samples, with the on-chip extraction utilizing a new method of eluate removal from the chip. Statistical significance was found by multiple unpaired *t*-tests (α = 0.05). Sample 5, *p* = 0.0029, and all others *p* > 0.222.

This new protocol was used, and the samples were analyzed with qPCR was run on the samples to evaluate their C_t_ values in Figure 10. Each sample was from the same stock solution of Heparin anticoagulated blood diluted to a concentration of 0.5 ng/μL. The C_t_ values off-chip and on-chip are closer in value compared to those in Figure 8. In Figure 8, as mentioned previously, some samples were omitted due to lack of amplification. All samples were included in Figure 10, which further shows the repeatability of this updated protocol.

### Final On-Chip Protocol

**FIGURE 11:**
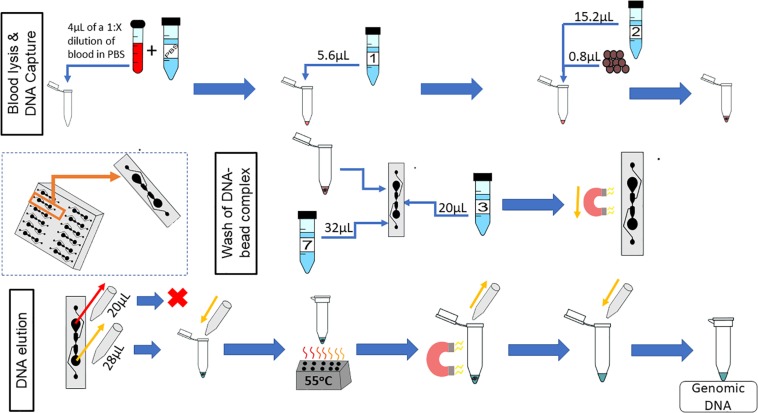
On-chip workflow. The microfluidic chip has 12 microfluidic separators; the protocol described is for one of these microfluidic separators. Whole blood is diluted 1:X with PBS, where X is dependent upon the age of the blood sample; 4 μL of this dilution is used for the on-chip protocol. Next, 5.6 μL of Lysis Buffer 1 is added to the diluted blood, mixed, and incubated for 5 min at room temperature. Subsequently, 0.8 μL of magnetic beads and 15.2 μL of Binding Buffer 2 are added to the lysed blood cells, and the combination is mixed and incubated for 5 min at room temperature. During the second incubation, 20 μL of Wash Buffer 3 is added to the Primary Wash Buffer Loading well. Then, 23 μL of the sample (lysed blood, binding buffer, and magnetic beads) is added to the sample well simultaneously with 32 μL of Elution Buffer 7. A magnet moves the magnetic beads from the sample well to the elution well. To elute the DNA, 28 μL of the eluate is removed from the elution well simultaneously with 20 μL of the sample well contents to reduce the effects of diffusion. The 28μL sample is placed into a tube, which is placed onto a heat block at 55°C for 10 min. The tube is then placed onto a magnetic rack and the supernatant is reserved.

The complete on-chip protocol is shown in Figure 11. Whole blood is diluted 1:X, in PBS, where X is dependent upon the length of time the blood sample has been stored. The concentration of gDNA in blood decreases with increasing storage time, so a sample that has been stored for longer would need to be diluted less (Bulla et al., 2016). It is recommended that the sample be diluted to at least 1:10, but as shown here, fresher samples should be diluted by a higher dilution factor. The volumes of the protocol can vary, if key physical restraints are met (Table 3).

**TABLE 3:** Restraints of the microfluidic chip.

Parameter	Restraint
Input DNA amount	Below 4 ng, or 667 white blood cells
Reagent volumes	Scaled linearly with sample volume
Sample Well volume	Volume must be lower than that of the elution well
Elution Well volume	Volume must be higher than that of the sample well
Sample loading	Sample well and elution well contents must be loaded simultaneously
Eluate removal	Elution well and sample well contents must be removed simultaneously, leaving equal residual volumes in both wells

### Next-Generation Sequencing Preparation of gDNA Extracted On-Chip

**FIGURE 12:**
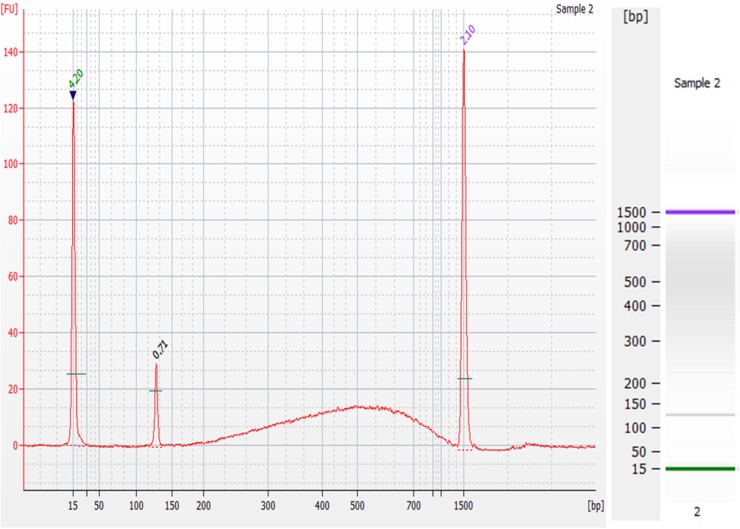
Preparation of gDNA extracted from the microfluidic chip for Next-Generation Sequencing. Electropherogram **(left)** and corresponding gel **(right)** of the DNA library prepared from an on-chip washed sample of gDNA using the NEXTFLEX^®^ Rapid DNA-Seq Kit. The peaks at 15 bp and 1500 bp are the upper and lower markers, the peak at 120 bp range is an adapter dimer, and the “bump” seen in the 200–1500 bp is the prepared library.

The goals of this device were to reduce the cost and time of gDNA extraction from whole blood as well as yielding gDNA that is of a quality suitable for NGS. To show the quality of the gDNA extracted, an on-chip washed sample that contained approximately 0.24 ng of gDNA was prepared for NGS using the 1-ng preparation protocol of the NEXTFLEX^®^ Rapid DNA-Seq Kit. This kit produces libraries for Illumina^®^ sequencing. The electropherogram and corresponding gel of the DNA library are shown in Figure 12. The prepared sample’s electropherogram appears as it should do according to the manufacturer’s expected results. The peaks at 15bp and 1500bp are the upper and lower markers, which are specific DNA sequences that are a part of the Agilent DNA 1000 kit, used to guide the sizing of the input sample. The peak at 120bp is an adapter dimer, which was expected, as excess adapter was added to exaggerate the results. The prepared library is the “bump” seen in the 200-1500bp range, which contains PCR-amplified fragmented gDNA sequences with the necessary modification for Illumina^®^ platforms.

## Conclusion

Genetic epidemiological studies aim to understand the role of genetics in disease etiology. These studies require analysis of multiple patient samples so as to obtain more conclusive measures of association. High-quality and pure DNA samples are needed, which involves complex sample preparation protocols. These protocols are successful for gDNA extraction using macroscale volumes and multiple washes, but there is still limited knowledge on the quantitative extraction efficiency in microscale geometries. Here, a microfluidic chip was designed that reduces the number of wash steps necessary for genomic DNA extraction from whole blood. Six extractions can be performed simultaneously, increasing the throughput of sample collection. The volume of blood needed is less than 1 μL (undiluted), the total volume of reagents needed is 75 μL, and the entire protocol takes approximately 40 min to extract gDNA from six samples.

The microfluidic chip designed here has the potential to reduce sample processing time and shipping costs associated with genetic epidemiological studies. When compared to similar technologies (Duarte et al., 2010; Gong and Li, 2014; Hung et al., 2015), though the blood is diluted, the gDNA yield is similar. Additionally, the yield of ∼3 ng is sufficient for NGS preparation, which can have an input of ∼1 ng. The device does not use electricity or complicated equipment, which increases its accessibility for a variety of settings. Even though we are still at early an stage of technology development, we believe that this fundamental understanding of molecular diffusional analysis and bead transport phenomena will provide insightful guidance for point-of-care biological diagnostic platforms that use small volumes of whole blood, such as from finger pricks. There are opportunities for optimization of the reaction components, such as increasing magnetic bead volumes and adjusting buffer ratios. Future directions of this device include the design of more specific assays targeting genes of interest and integration of an on-chip heated elution step to further reduce manual steps. Additionally, an advantage using of magnetic beads is that it allows for further automation of the protocol. Overall, this microfluidic chip is simple to use while resulting in high-quality DNA samples, which can increase the throughput of genetic epidemiological studies.

## Data Availability Statement

The raw data supporting the conclusions of this article will be made available by the authors, without undue reservation, to any qualified researcher.

## Author Contributions

AT was responsible for project administration and supervision. KL conceptualized the project, performed the experiments, collected and analyzed the data, and wrote the original draft of the manuscript. Both authors were responsible for editing subsequent drafts of the manuscript.

## Conflict of Interest

The authors declare that the research was conducted in the absence of any commercial or financial relationships that could be construed as a potential conflict of interest.

## Supplementary Material

FIGURE S1 **(A)** The PDMS-glass microfluidic chip. **(B)** The aluminum mold. **(C)** The sandwich mold composed of the SU-8 master mold, uncured PDMS, and the aluminum mold.

FIGURE S2 To model the magnetic force felt by the glass slide, and hence the paramagnetic beads, COMSOL Multiphysics was used. With an air box, the magnet is on the bottom, above it is the glass slide that the microfluidic chip would be mounted upon, and above the glass slide is a mock magnetic bead (for visual purposes). The input for the magnet was the remnant flux density, given by the manufacturer for the N42 magnet as 13,200 Gauss and for their strongest magnet (N52) this value is 14,800 Gauss. The image depicts the magnetic flux density in Tesla. Force calculations revealed that the glass slide feels a force of 37.1N with a N42 magnet and 46.7N with a N52 magnet.
