# Supplementary figures and images for: Parallel DNA Extraction From Whole Blood for Rapid Sample Generation in Genetic Epidemiological Studies
Source: Front Genet. 2020 Apr 29;11:374. doi: 10.3389/fgene.2020.00374 (PMC7201099; doi:10.3389/fgene.2020.00374)

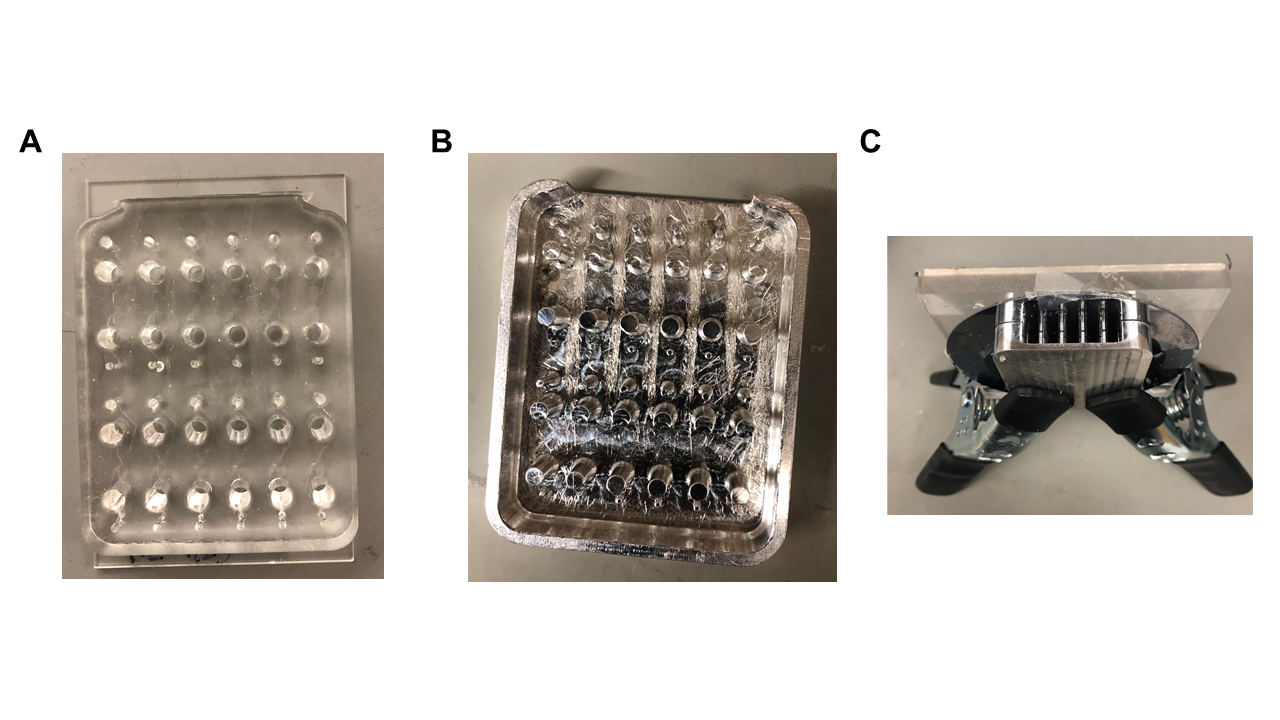

Supplement: FIGURE S1 — (A) The PDMS-glass microfluidic chip. (B) The aluminum mold. (C) The sandwich mold composed of the SU-8 master mold, uncured PDMS, and the aluminum mold. [file Image_1.tif]

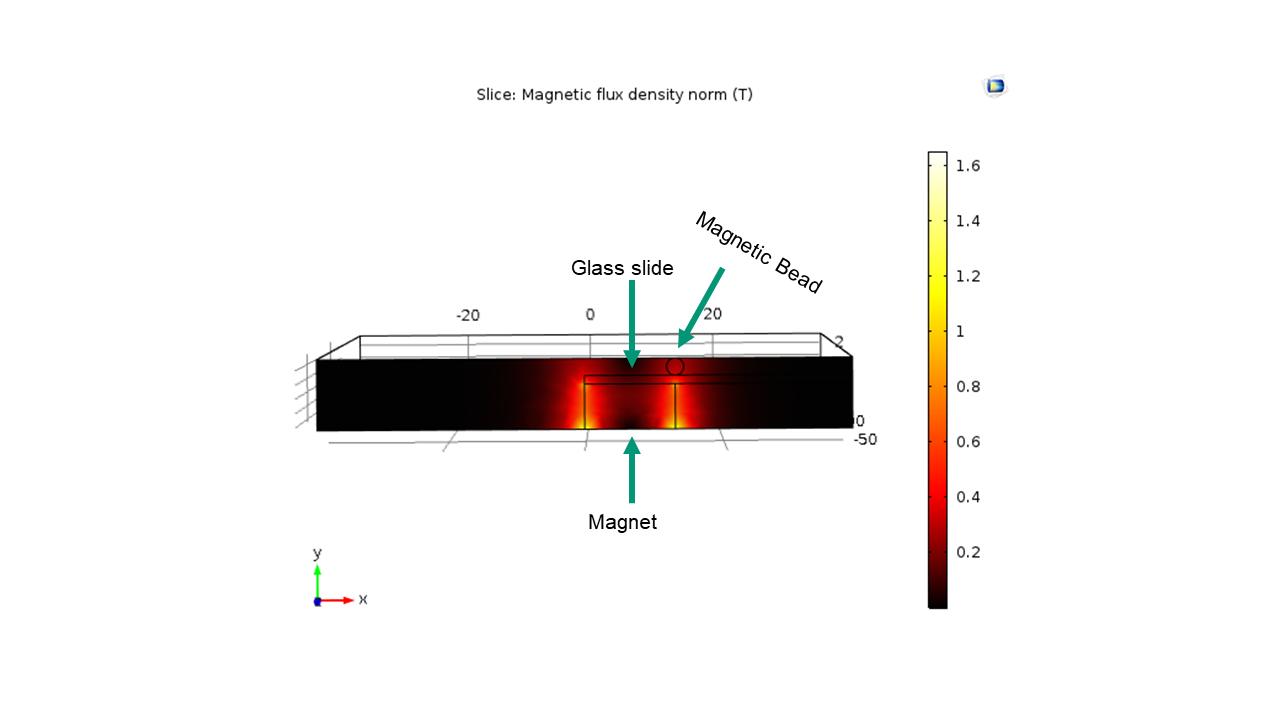

Supplement: FIGURE S2 — To model the magnetic force felt by the glass slide, and hence the paramagnetic beads, COMSOL Multiphysics was used. With an air box, the magnet is on the bottom, above it is the glass slide that the microfluidic chip would be mounted upon, and above the glass slide is a mock magnetic bead (for visual purposes). The input for the magnet was the remnant flux density, given by the manufacturer for the N42 magnet as 13,200 Gauss and for their strongest magnet (N52) this value is 14,800 Gauss. The image depicts the magnetic flux density in Tesla. Force calculations revealed that the glass slide feels a force of 37.1N with a N42 magnet and 46.7N with a N52 magnet. [file Image_2.tif]
